# Assessment of Cardiac Autonomic Function in Relation to Methylmercury Neurotoxicity

**DOI:** 10.3390/toxics6030038

**Published:** 2018-07-20

**Authors:** Kanae Karita, Toyoto Iwata, Eri Maeda, Mineshi Sakamoto, Katsuyuki Murata

**Affiliations:** 1 Department of Hygiene and Public Health, Kyorin University School of Medicine, Mitaka, Tokyo 181-8611, Japan; kanae@ks.kyorin-u.ac.jp; 2 Department of Environmental Health Sciences, Akita University Graduate School of Medicine, Akita, Akita 010-8543, Japan; iwata@med.akita-u.ac.jp (T.I.); erimaeda@med.akita-u.ac.jp (E.M.); 3 National Institute for Minamata Disease, Minamata, Kumamoto 867-0008, Japan; sakamoto@nimd.go.jp

**Keywords:** heart rate variability, methylmercury neurotoxicity, review, sympathodominant state

## Abstract

After the European Food Safety Authority reviewed reports of methylmercury and heart rate variability (HRV) in 2012, the panel concluded that, although some studies of cardiac autonomy suggested an autonomic effect of methylmercury, the results were inconsistent among studies and the implications for health were unclear. In this study, we reconsider this association by adding a perspective on the physiological context. Cardiovascular rhythmicity is usually studied within different frequency domains of HRV. Three spectral components are usually detected; in humans these are centered at <0.04 Hz, 0.15 Hz (LF), and 0.3 Hz (HF). LF and HF (sympathetic and parasympathetic activities, respectively) are evaluated in terms of frequency and power. By searching PubMed, we identified 13 studies examining the effect of methylmercury exposure on HRV in human populations in the Faroe Islands, the Seychelles and other countries. Considering both reduced HRV and sympathodominant state (i.e., lower HF, higher LF, or higher LF/HF ratio) as autonomic abnormality, eight of them showed the significant association with methylmercury exposure. Five studies failed to demonstrate any significant association. In conclusion, these data suggest that increased methylmercury exposure was consistently associated with autonomic abnormality, though the influence of methylmercury on HRV (e.g., LF) might differ for prenatal and postnatal exposures. The results with HRV should be included in the risk characterization of methylmercury. The HRV parameters calculated by frequency domain analysis appear to be more sensitive to methylmercury exposure than those by time domain analysis.

## 1. Introduction

The measurement of heart rate variability (HRV; or, the coefficient of variation of the R‒R intervals, CV_RR_) using frequency domain analysis is an effective approach for the objective assessment of the autonomic nervous function [1,2,3]. In a seminal study, Wheeler and Watkins observed a striking reduction or absence of beat-to-beat variation during both quiet and deep breathing in diabetic patients with autonomic neuropathy [4]. In 2012, the European Food Safety Authority (EFSA) reviewed several reports examining the influence of methylmercury on HRV and concluded that, although some studies of cardiac autonomy suggested an autonomic nervous effect of methylmercury, the results were inconsistent across studies and the implications for health were unclear [5]. Gribble et al. [6] reached a similar conclusion, finding that a major limitation of studies examining the influence of methylmercury on HRV was a lack of standardized methods for performing and reporting HRV measurements. Concerning the implications of HRV findings for health, however, the existing data may allow other interpretations besides those drawn by the EFSA panel. This study was intended to reconsider the involvement of methylmercury in HRV by adding a perspective on the physiological context.

## 2. Materials and Methods

### 2.1. Data Sources and Extraction

We searched for published papers using a set of keywords (“mercury,” “heart rate variability,” and “humans”) in PubMed, US National Library of Medicine, and identified 33 citations as of 31 January 2018. We excluded review papers (*n* = 6), case series (*n* = 3), studies addressing occupational mercury exposure (*n* = 2) or studies in which data for either HRV (*n* = 3) or mercury biomarkers (*n* = 6) were not described. Finally, included were 13 studies of methylmercury exposure and HRV: ten studies [7,8,9,10,11,12,13,14,15,16] that were included in a previous systematic review by Gribble et al. [6] and three new studies [17,18,19]. In all of the identified studies, the statistical significance was set at *p* < 0.05.

### 2.2. Physiological Background

The autonomic nervous system innervates every organ in the body, and its neural organization in the brain, spinal cord, and periphery is as complex as the somatic nervous system [1]. A vasomotor center, located in the medulla oblongata, and vagal cardioinhibitory neurons, located primarily within the ventrolateral subdivision of the nucleus ambiguus, are thought to be especially important in the regulation of the cardiovascular system. The autonomic nervous system plays a role in triggering or sustaining malignant ventricular arrhythmias. Higher sympathetic activity, unopposed by vagal activity, promotes arrhythmia through a variety of mechanisms such as reducing the ventricular refractory period and the ventricular fibrillation threshold, promoting triggered activity afterpotentials, and enhancing automaticity [20]. By contrast, vagal stimulation opposes these changes and reduces the effects of sympathetic stimulation by prolonging refractoriness, elevating the ventricular fibrillation threshold, and reducing automaticity. For this reason, HRV testing is important for assessing cardiac autonomic function in clinical applications because of the availability of low-cost and non-invasive methods.

**Figure 1:**
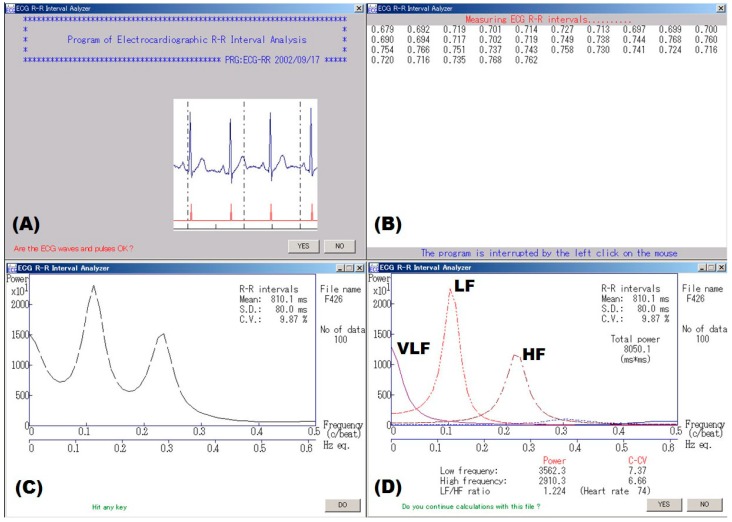
An example of spectral analysis of ECG R‒R intervals. (**A**) An examiner confirmed waveforms on the electrocardiogram. (**B**) Electrocardiographic 300 R‒R intervals or 5-min R‒R intervals were measured in real time. (**C**) Spectral analysis using autoregressive model was made for consecutive 100 R‒R intervals with the minimal standard deviation (SD) that were automatically extracted from the obtained data, and (**D**) component analysis was made for the data. Finally, a coefficient of variation (C.V. or CV_RR_) was calculated from the mean and SD of the same data. VLF, LF, and HF represent very low frequency, low frequency and high frequency bands, respectively.

The procedure for data sampling and spectral analysis of successive R‒R intervals on electrocardiograph (ECG) is illustrated in Figure 1. The sinus rhythm shows fluctuation around the mean R‒R interval (or heart rate) due to continuous changes in the sympathovagal balance [21]. Rhythmicity is usually studied within different frequency domains. Three major spectral components, calculated by frequency domain analysis using a fast Fourier transform (FFT) or autoregressive model, are usually detected. In humans, these are centered at a very low frequency (VLF—below 0.04 Hz), a low frequency (LF—around 0.15 Hz) and a high frequency (HF—around 0.3 Hz) [1]. Spectral analysis involves subjecting a time series of R‒R intervals to a mathematical transformation which separates those R‒R intervals into individual harmonics which are identifiable through their discrete frequencies. The LF and HF are evaluated in terms of frequency and amplitude; the latter commonly assessed by its area (i.e., power spectral density or colloquially “power”). The VLF has been equated with a thermoregulatory or vasomotor influence, the LF with baroreflex control and arterial pressure variations, and the HF with respiration [1,21]. Therefore, the LF and HF are thought to be mediated by sympathetic and parasympathetic pathways, respectively [1,2]. The CV_RR_ was defined as the ratio of the standard deviation (SD) of the R‒R intervals to the average value (RR_mean_). Likewise, the CV_LF_ and CV_HF_ were defined as the ratios of the square roots of each component power spectral density (i.e., LF and HF) to the RR_mean_, which can be compared between different populations because they are adjusted for the mean R‒R interval of each subject [3]. In addition, the LF/HF ratio and %LF (= LF/(LF + HF) × 100, %) are used as HRV parameters for frequency domain metrics. 

### 2.3. Interpretation of HRV Parameters

Autonomic abnormality is thought to represent a sympathodominant state of the sympathovagal balance during the initial stages and depressed HRV at the severe stage. The latter manifests as a reduction in total (e.g., CV_RR_) and in specific power (i.e., HF, LF, CV_HF_, and CV_LF_) of spectral components and is observed in patients after acute myocardial infarction [2], and those with autonomic neuropathy due to diabetes mellitus [1] or alcoholism [22]. The sympathodominant state has three patterns compared with healthy controls: (i) lower HF (with no significant difference in LF); (ii) higher LF (with no significant difference in HF); and (iii) higher LF/HF ratio (or %LF). Previous studies on the effects of occupational and environmental factors on HRV parameters have primarily demonstrated a lower HF pattern (due to lead, styrene, mixed solvents such as *n*-hexane and toluene; local vibration in chain-saw workers [23,24,25,26,27,28,29,30]; exposure to sarin [31]; and long commuting times of 90 min or more [32]). In addition, Pagani et al. [33] observed a higher LF at rest in patients diagnosed with chronic fatigue syndrome in comparison with healthy control subjects (73 ± 11 and 51 ± 10 normalized units, respectively, *p* < 0.05), but responsiveness to mental stimuli (mental arithmetic) was reduced in the patients compared with the controls. Thus, the above interpretation may be applicable for HRV assessment in a static state, but not in an active mode, because the latter cannot preserve stationarity of autonomic modulations.

## 3. Results

### 3.1. Relations of Methylmercury to HRV

Table 1 shows the characteristics of epidemiological studies examining the effects of methylmercury exposure on HRV in chronological order, together with the range of exposure levels (i.e., minimum and maximum) for the studies that provided this information. In one case-control study, patients officially certified as having fetal-type Minamata disease (methylmercury poisoning due to *in utero* exposure) showed significantly reduced HF compared to age- and sex-matched healthy controls, but no data on prenatal or postnatal methylmercury exposure were included [7].

In the Faroese birth cohort study, cord-blood mercury was associated with decreased LF and HF in children aged 14 years [8], and there were significant associations between increased mercury levels in cord blood and hair at 7 years and decreased LF and CV_LF_ in the children aged 7 years. In a retrospective cohort study using dry cord tissue, methylmercury levels in cord tissue were associated with increased LF/HF ratio and decreased HF in Japanese children at 7 years of age [9]. The median mercury level in this population was estimated to be 2.24 (range, 0.43–9.26) µg/g in maternal hair at parturition according to the equation of Akagi et al. [34]. In 11-year-old Inuit children, blood mercury was associated with decreased CV_RR_ (adjusted *β* = −0.06, *p* = 0.01) and LF (adjusted *β* = −0.24, *p* = 0.02), though neither cord-blood mercury nor hair mercury at 11 years was significantly associated with any HRV parameter [16]. The Seychelles child development study found no significant associations between prenatal or postnatal exposure to methylmercury and HRV parameters [17].

In a cross-sectional study, Lim et al. [13] reported that hair mercury was negatively related to HF in Korean adults (*p* < 0.05). In Cree adults, blood and hair mercury levels were positively related to LF/HF ratio, LF, and HF (*p* < 0.01) [14]. Similarly, in French Polynesians aged 12–17 years, significant differences were observed in LF/HF ratio, LF, and HF between the second (7.9–10.0 µg/L) and third (11.0–26.0 µg/L) tertiles of blood mercury concentration [15]. Among Faroese whaling men, blood mercury level was associated with increased CV_RR_, CV_HR_, and CV_LF_, but latent mercury level, estimated from mercury levels in blood, toe nail, and hair (7 years ago) using a structural equation model, was not significantly associated with any HRV parameter [11]. In Inuit adults, blood mercury was significantly correlated with CV_RR_ and LF (*r* = −0.18 and *r* = −0.18, respectively), but these significant associations disappeared after adjusting for potential confounders [10]. In avid fish consumers, either blood total mercury or serum docosahexaenoic acid (DHA) and eicosapentaenoic acid (EPA) level was not significantly associated with any HRV parameter in a multiple regression analysis [19]. Gump et al. [18] measured LF, HF, and LF/HF ratio at rest and during stress in children aged 9–11 years to assess parasympathetic responses to acute stress, but neither blood mercury nor lead was significantly related to baseline HRV parameters (HRV data not shown).

An intervention study reported that methylmercury exposure of 3.4 µg/kg body weight/week for 14 weeks via fish consumption induced a temporary sympathodominant state (*p* = 0.014 for higher CV_LF_; *p* = 0.076 for elevated LF/HF ratio) [12]. Also, age-, sex-, and body mass index-adjusted CV_LF_ was positively related to hair mercury at the 15th week (*p* < 0.001), though such significant relations were not observed at baseline or at the 29th week of follow-up. Table 2 presents a summary of studies examining the association between mercury levels and HRV parameters. A detailed discussion of these results follows in Section 4.1.

### 3.2. Measurement of HRV

HRV has been analyzed using a Holter monitoring system [10,14,15,16,19], an ECG analyzer with analog-to-digital converter [7,8,9,12], or other ECG measuring instruments [11,13,17,18]. ECG signals for R‒R intervals were digitalized at 128 Hz [10,14,15,16], 200 Hz [17], 250 Hz [7], 500 Hz [18], and 1000 Hz [8,9,12]. Three reports did not mention the sampling frequency [11,13,19]. Most of the study subjects were examined in a supine position [7,8,9,11,12,17] after subjects lay quietly for two min or longer, but the subjects examined by Lim et al. [13] were sitting in a quiet and dark room. The remaining reports did not provide any detailed information [10,14,15,16,18,19].

**Figure 2:**
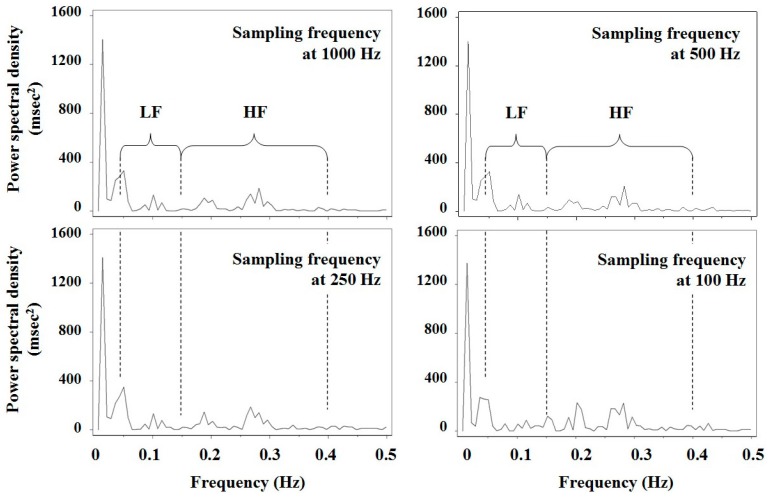
Results of spectral analysis of 128 consecutive R‒R intervals using a fast Fourier transform in a male student. The original R‒R intervals were measured at the sampling frequency of 1000 Hz after a subject rested in the supine position for 10 min, and data for lower sampling frequencies (500 Hz, 250 Hz and 100 Hz) were generated from the original data taking into account random error. LF and HF represent low frequency and high frequency bands, respectively.

To examine the effect of sampling frequency on LF and HF, 128 consecutive R‒R intervals with the minimal SD in 61 male students aged 18–26 years [35] were reanalyzed using FFT spectral analysis, as shown in Figure 2. The original R‒R intervals were measured at a sampling frequency of 1000 Hz after each subject rested in a supine position for 10 min, and data for lower sampling frequencies (500 Hz, 250 Hz, 200 Hz, 125 Hz, and 100 Hz) were generated from the original data taking into account random error. Table 3 presents the HRV parameters calculated by the spectral analysis. All the HRV parameters were significantly different among six frequency-band groups. In particular, most of significant differences were observed between sampling frequency bands of less than 200 Hz and 200–1000 Hz.

## 4. Discussion

### 4.1. Assessment of Methylmercury Neurotoxicity

With regard to the interpretation of HRV at rest, depressed HRV (e.g., CV_RR_ of less than 2%) takes precedence of a sympathodominant state because it indicates cardiac autonomic hypofunction while other readouts reflect a result of disrupted sympathovagal balance [21,36]. Empirically, reduced HF precedes elevated LF and LF/HF ratio, as mentioned in Section 2.3. Furthermore, elevated LF may precede high LF/HF ratio because the ratio is a relative measure of sympathetic and parasympathetic nerve activities and is not always suggestive of autonomic dysfunction [37]. Of course, data that did not achieve statistical significance (c(±) or r(±)) are no longer discussed because any marginal association was likely attributable to chance. In light of these criteria, some commonalities can be observed across the 13 studies examined here, though Gribble et al. [6] judged the above evidence was too limited to draw causal inferences. Namely, increased mercury levels were associated with autonomic abnormality as shown in Table 2 (especially, gray areas). In addition, some fetal-type and child Minamata disease patients showed vegetative symptoms including dizziness, orthostatic syncope, palpitation, breathlessness, and nausea [38], along with hypersalivation and ileus [7].

Of the eight studies showing significant associations in Table 2, three demonstrated a potential causal link between prenatal exposure level of methylmercury and autonomic hypofunction [7,8,9]; this finding is similar to some results for cognitive deficits [39,40] and mental retardations [41]. Likewise, five studies suggested a significant relationship existed between postnatal exposure levels and HRV parameters analyzed using the frequency domain method. It is relatively straightforward to infer causal relations from cohort studies [8,9] and an intervention study [12]. Contrariwise, it is difficult to discriminate the effects of postnatal exposure to methylmercury from those of prenatal exposure based on cross-sectional studies with no information about prenatal exposure levels [13,14,15], inasmuch as there is evidence that mercury levels at 7 years of age reflect the prenatal exposure levels to some extent [42]. In cases of this nature, it would be necessary to develop a plan estimating prenatal exposure levels: For example, current mercury levels in hair of mothers, who had not changed their dietary habits, might be used as a proxy for mercury exposure during pregnancy [43,44]. This method cannot be applicable to subjects aged more than 7 years, as mentioned above. Insignificant findings may have been attributable to extremely low exposure levels [18,19], measurements of different HRV parameters [17] or subjects more than 45 years of average age [10,11,15,19].

Only one report seemed to show complicated findings [8]. In this study, cord blood mercury was associated with reduced CV_RR_, CV_HF_, HF, and LF in 14-year-old children, but the 7-year-old children showed only a significant association between cord blood mercury and lower LF. There are at least two possible explanations for this paradox. First, since hair mercury levels at 7 years were higher than those at 14 years (Table 1), the effect of prenatal methylmercury exposure on HRV may have been distorted by postnatal exposure. Thereafter, as postnatal exposure levels decreased with age, the influence of the prenatal exposure may have become predominant in the 14-year-old children. In support of this possibility, patients with fetal-type Minamata disease showed autonomic hypofunction approximately 45 years after the onset of the disease [7]. Second, short sleep duration in preschool children aged 5 and 6 years was associated with reduced HRV [45,46]; children who slept less than 10 h per weekday showed significantly lower CV_RR_, CV_LF_, CV_HF_, LF, and HF than those who slept 10 h per weekday or more, but no significant difference in LF/HF ratio was observed between the two groups. By contrast, in 150 students aged 18–26 (mean 20) years, weekday sleep duration was not significantly associated with any HRV parameter [35]; whereas, white-collar workers with commuting times of 90 min or more showed decreased CV_HF_ compared to those with commuting time of 60–89 min or less than 60 min (2.10 ± 1.17%, 2.25 ± 1.10%, 2.58 ± 1.33%, respectively) [32]. Thus, it is possible that sleep duration may have confounded the HRV data for the 7-year-old children. Regrettably, the Faroes study did not examine the participants’ sleep durations. Taken together, the data suggest that prenatal methylmercury exposure consistently affected HRV parameters in response to the exposure dose, i.e., ranging from a sympathodominant state to autonomic dysfunction. Nevertheless, HRV parameters are susceptible to physiological conditions such as sleep duration in young children [45,46] and mental stimuli [33], in addition to recent methylmercury exposure at relatively high levels.

The Seychelles child development study, which used time domain metrics, failed to find a significant association between hair mercury levels and HRV in a cohort of 19-year-olds [7]. Likewise, Faroese whaling men showed no significant associations between indicators of latent exposure to methylmercury and HRV parameters [11]. In subjects of these studies, recent exposure levels (total mercury in hair) were considerably higher (mean 9.5 µg/g for the former study and geometric mean 7.31 µg/g for the latter study) than those in the general population of other countries. Thus, comparison of HRV parameters between high/frequent and low/infrequent fish consumers should probably have been made. Autonomic function may have been affected by prenatal methylmercury exposure (though not so drastically as fetal-type Minamata disease patients [7]) and recent exposure levels, different from prenatal ones, may have changed greatly due to diversity in habits (for instance, consumption of methylmercury-contaminated fish). In two studies examining the same HRV parameters, the CV_RR_ and CV_HF_ of the above Faroese whaling men were 2.99% and 1.30%, respectively, which were lower than those of 23 Japanese healthy men aged 30–63 (mean 49) years (3.75% and 1.79%, respectively) [22]. The Seychelles child development study did not employ comparable HRV parameters. Thus, not only dose-effect relationships but also comparison between subgroups of the study population should have been attempted during the data analysis, as Varela and coworkers did [15].

In reports demonstrating a significant relationship between methylmercury exposure and HRV parameters, prenatal mercury levels showed a geometric mean 4.22 µg/g [8] and an equivalent median of 2.24 µg/g [9] in maternal hair at parturition; postnatal mercury levels were 0.83 µg/g in hair [13], 5.7 µg/L in blood [14] and 2.9 µg/L in blood [16] at the time of testing, whereas two reports except one [16] did not describe prenatal exposure levels. It would not be straightforward to estimate the critical concentration of methylmercury from these data. Valera et al. [15] and Yaginuma-Sakurai et al. [12] observed significant differences in HRV parameters between comparable subgroups with different mercury levels; specifically, the latter study indicated that a 14-week methylmercury exposure caused significant changes in some HRV parameters. Taken together, the data suggest that the average mercury level reported in the study by Yaginuma-Sakurai et al. [12] (8.76 µg/g in hair) is a reasonable estimate of a critical dose of total mercury likely to affect HRV. The EFSA panel regarded the point of departure (POD) as 11.5 µg/g for maternal mercury levels in hair and 46 µg/L for those in blood by applying a hair-to-blood ratio of 250 [5]. Since the value of 8.76 µg/g in hair corresponds to 35 µg/L in blood, the results suggest a reference dose ranging from 10 to 20 µg/L in blood after taking uncertainty factor into account; whereas, the average mercury level in whole blood of the same subjects measured after the 14-week exposure was 26.9 µg/L [47]. In such cases, intake of *n*-3 polyunsaturated fatty acids (PUFA) and selenium resulting from fish consumption should be considered in each country [12,48,49,50].

The effect of methylmercury on cardiac autonomic function was suggested to be reversible by an intervention study [12], separately from a cohort study by Grandjean et al. [8]. The adverse effects in the former study disappeared after 14-week wash-out period following cessation of the exposure. Recent mercury levels in hair of Faroese children aged 14 years were associated with a prolonged latency between the pons and midbrain of the auditory pathway [51], and the brainstem auditory evoked potential latency showed clear negative associations with LF and CV_LF_ [8]. Moreover, all the HRV parameters were largely reduced in comatose children with brainstem dysfunction [52], implying that impairment in the higher center of cardiac autonomic function can lead to decrease both in HF and LF power. Therefore, the pathology of autonomic imbalance due to methylmercury appears to differ for prenatal and postnatal exposures, hypothesizing that prenatal methylmercury exposure can readily impair the higher center of cardiac autonomic function and the postnatal exposure does the same in the periphery. In this case, the directionality of LF would differ, with reduced LF for prenatal exposures [8] and elevated LF for postnatal exposure [9,12,14,15].

### 4.2. Factors Affecting the Assessment of Cardiac Autonomic Function

The Task Force of the European Society of Cardiology and the North American Society of Pacing and Electrophysiology recommended either a 5-min recording for frequency domain metrics or a 24-h recording for time domain metrics [2]. The latter is useful for clarifying the pathophysiology of cardiac autonomic function in clinical medicine—however, whether it is useful for toxicological studies in human populations remains disputable. It would be not feasible to examine a large number of subjects with 24-h monitor, and for time domain parameters, the longer the R‒R interval sample, the greater the natural variation of the signal due to heterogeneous influences on heart rate [1]. Moreover, time domain variables provide no information about the sympathetic activity [2]. None of the studies in this review used 24-h ECG monitoring. Instead, posture during measurement of R‒R intervals and sampling frequency in digitizing ECG signals differed among these studies. Subject posture during measurement (supine rest, upright tilt, or sitting) affects the LF/HF ratio and the power of each component directly [2,53], and spectral signal is readily distorted by movement artifacts and ectopic beats [1]. For this reason, it is important to retain posture across measurement. Valera et al. [10,14,15,16], using ambulatory 2-h Holter monitoring, excluded R‒R intervals whose duration was less than 80% or more than 120% of the running R‒R average before performing frequency domain analysis; therefore, their data might not represent a time series of successive R‒R intervals. In addition, as Table 3 shows, the data precision of R‒R intervals depends on the sampling frequency [2,54]. A sampling frequency of 200 Hz or higher, in disagreement with the report by Merri et al. [54], appears to be required to preserve measurement precision. Clearly, research in human toxicology will not progress unless researchers employ comparable (and, if possible, R‒R interval-adjusted) endpoints for HRV such as the CV_RR_, CV_HF_, and CV_LF_.

When assessing a causal influence on HRV in humans, many potential confounders (age, sex, drinking and smoking habits, sleep duration, and mental stimuli) should be considered [3,55,56]. In the data analysis, since it is difficult to control for the effect of age in subjects at an extremely wide range of age (e.g., 5-83 years old [13]), such analyses have to be made in some age-specific groups separately. Likewise, consumption of *n*-3 PUFA such as DHA and EPA is suggested to affect HRV parameters [6,12,57], while body mass index and body fat percentage seem to be associated with corrected Q‒T (QTc) intervals on ECG and heart rate, but not with HRV parameters [35]. Several disorders have been suggested to affect HRV parameters, including: Acute myocardial infarction, congestive heart failure, coronary artery disease, coronary atherosclerosis, myocardial dysfunction, cardiac transplantation, Shy-Drager syndrome, Parkinsonism, Guillain-Barre syndrome, tetraplegia, spinocerebellar degeneration, diabetic neuropathy, renal failure, chronic alcoholism, and essential hypertension [2,3]. In addition, although levels of environmental pollutants such as PCBs and lead are low in developed countries [58], concurrent exposure models would be necessary to consider the interactive effects of substances other than methylmercury [59,60]. For that reason, special attention should be paid to subjects with such disorders, as well as potential confounders and exposure to other neurotoxic substances, when conducting toxicological studies in humans.

Apart from HRV, using spectral analysis, QTc intervals are frequently used as another method for assessing autonomic nervous function [35,61,62,63,64,65,66], inasmuch as Q‒T interval on ECG represents the duration between ventricular depolarization and subsequent repolarization [67]. QTc prolongation is suggested to be associated with elevated risk of heart disease and sudden cardiac death [68,69,70,71]. Therefore, the QTc interval may be a more promising indicator than HRV parameters for investigating the pathophysiology of cardiovascular events involved in methylmercury exposure, though it was not significantly associated with prenatal or postnatal methylmercury exposures at relatively low levels [9,19].

## 5. Conclusions

The HRV parameters analyzed using the frequency domain method, as well as CV_RR_, appear to be more sensitive to methylmercury exposure than those using the time domain method. Most of the studies addressed in this review suggested that increased mercury levels were associated with autonomic dysfunction including a sympathodominant state, though the effect of methylmercury on HRV (e.g., LF) might differ for prenatal and postnatal exposures. In an intervention study carried out by Yaginuma-Sakurai et al., a significant difference in LF analyzed using spectral analysis was observed between the experimental group (mean mercury levels of 8.76 µg/g in hair and 26.9 µg/L in blood) and control group. Exposures near this dose may have critical effect on cardiac autonomic function. Therefore, the POD to inform a new tolerable weekly intake should be based on this critical concentration because it is lower than that established by the EFSA panel (11.5 µg/g in maternal hair). This result could probably be applicable for the general population, including pregnant women and unborn children.

## Figures and Tables

**Table 1:** Human studies addressing the effects of methylmercury exposure on heart rate variability.

Authors (Year) [Ref #]	Place	Subjects	Prenatal Exposure (Total Mercury Levels) *	Postnatal Exposure (Total Mercury Levels)
Oka et al. (2003) [7]	Minamata, Japan	9 FMD patients and 13 controls		
Grandjean et al. (2004) [8]	Faroe Islands, Denmark	857 children aged 7 years	GM 22.6 µg/L, IQR 13.2~40.8 µg/L in cord blood; GM 4.22 µg/g, IQR 2.55~7.68 µg/g in maternal hair	GM 2.99 µg/g, IQR 1.69~6.20 µg/g in hair
		857 children aged 14 years		GM 0.96 µg/g, IQR 0.45~2.29 µg/g in hair
Murata et al. (2006) [9]	Japan	136 children	Med 0.089 µg/g, range 0.017~0.367 µg/g in cord tissue	Med 1.66 µg/g, range 0.43~6.32 µg/g in hair
Valera et al. (2008) [10]	Nunavik, Canada	205 Inuit adults		GM 19.6 µg/L, range 0.5~152 µg/L in blood
Choi et al. (2009) [11]	Faroe Islands, Denmark	42 whaling men		GM 7.31 µg/g, IQR 4.52~13.4 µg/g in hair; GM 29.5 µg/L, IQR 18.7~46.1 µg/L in blood
Yaginuma-Sakurai et al. (2010) [12]	Sendai, Japan	Intervention group (IG): 27 adults Control group (CG): 27 adults		IG: 2.30 ± 1.08 µg/g (Mean ± SD, 0th week), 8.76 ± 2.01 µg/g (15th week); CG: 2.27 ± 1.2 µg/g (0th week), 2.14 ± 1.03 µg/g (15th week) in hair
Lim et al. (2010) [13]	South Korea	1589 adults		GM 0.83 µg/g, IQR 0.56~1.28 µg/g in hair
Valera et al. (2011) [14]	Quebec, Canada	724 Cree adults		Med 5.7 µg/L, IQR 1.2~8.8 µg/L in blood
Valera et al. (2011) [15]	French Polynesia	101 teenagers		Med 8.5 µg/L, IQR 6.3~11.0 µg/L in blood
		180 adults		Med 13.5 µg/L, IQR 8.5~22.0 µg/L in blood
Valera et al. (2012) [16]	Nunavik, Canada	226 Inuit children	Med 16.3 µg/L, IQR 9.0~28.0 µg/L in cord blood	Med 2.9 µg/L, IQR 1.5~5.6 µg/L in blood
Periard et al. (2015) [17]	Seychelles	95 adolescents	Mean 6.7 µg/g, range 0.7~21.3 µg/g in maternal hair	Mean 9.5 µg/g, range 2.0~28.1 µg/g in hair
Gump et al. (2017) [18]	Syracuse, NY, USA	203 children		Mean 0.4 µg/L, range 0.01~11.65 µg/L in blood
Miller et al. (2017) [19]	Long Island, NY, USA	94 fish consumers		8.4 ± 8.6 (Mean ± SD) µg/L in blood

* Methylmercury levels were measured only in cord tissue [26]. *Abbreviations:* FMD, fetal-type Minamata disease; GM, geometric mean value; IQR, interquartile range (25th and 75th percentiles); Med, median value.

**Table 2:** Summary of associations between mercury levels and heart rate variability (HRV) parameters.

Authors (Year) [Ref #]	Mean Age at the Time of Examination	Exposure Period	HRV Parameters			
			CV_RR_	HF-Related Parameters	LF-Related Parameters	LF/HF Ratio
Oka et al. (2003) [7]	Patients 44.3 years, controls 42.9 years	prenatal	c(±)	c(‒)	c(±)	
Grandjean et al. (2004) [8]	7 years	prenatal	r(±)	r(±)	r(‒)	r(±)
		postnatal	r(±)	r(±)	r(‒)	r(±)
	14 years	prenatal	r(‒)	r(‒)	r(‒)	r(±)
		postnatal	r(±)	r(±)	r(±)	r(±)
Murata et al. (2006) [9]	6.9 years	prenatal		r(‒)	r(±)	r(+)
		postnatal		r(±)	r(+)	r (±)
Valera et al. (2008) [10]	52.1 years	postnatal	r(±)	r(±)	r(±)	r(±)
Choi et al. (2009) [11]	58.9 years	postnatal	r(±)	r(±)	r(±)	
Yaginuma-Sakurai et al. (2010) [12]	Intervention 25.2 years; control 23.7 years	postnatal	c(±)	c(±)	c(+)	c(±)
			r(±)	r(±)	r(+)	r(±)
Lim et al. (2010) [13]	33 years	postnatal		r(‒)	r(±)	
Valera et al. (2011) [14]	35 years	postnatal		r(+)	r(+)	r(+)
Valera et al. (2011) [15]	14.2 years	postnatal		c(‒)	c(+)	c(+)
	48.6 years	postnatal		c(±)	c(±)	c(±)
Valera et al. (2012) [16]	11.3 years	prenatal	r(±)	r(±)	r(±)	r(±)
		postnatal	r(‒)	r(±)	r(‒)	r(±)
Periard et al. (2015) [17]	19.5 years	prenatal		r(±)		r(±)
		postnatal		r(±)		r(±)
Gump et al. (2017) [18]	10.6 years	postnatal		r(±)	r(±)	r(±)
Miller et al. (2017) [19]	48.9 years	postnatal		r(±)	r(±)	r(±)

*Notes:* c(‒), significantly low in comparison; c(+), significantly high in comparison; c(±), not significant in comparison; r(‒), significantly negative relation; r(+), significantly positive relation; r(±), no significant relation. Gray areas show a sympathodominant state or autonomic hypofunction.

**Table 3:** Mean +/- SD values of HRV parameters measured at different sampling frequencies in 61 male students of reference [35].

Parameters	Sampling Frequency					
	1000 Hz	500 Hz	250 Hz	200 Hz	125 Hz	100 Hz
RR_mean_ (msec)	965.1 ± 162.9	965.1 ± 162.9	965.1 ± 162.9	965.1 ± 162.9	965.2 ± 162.9 *	965.2 ± 162.9
RR_SD_ (msec)	51.74 ± 21.35	51.75 ± 21.35	51.75 ± 21.34	51.75 ± 21.33	51.82 ± 21.29 *	51.80 ± 21.27 *
CV_RR_ (%)	5.321 ± 1.902	5.322 ± 1.903	5.323 ± 1.903	5.323 ± 1.902	5.330 ± 1.896 *	5.328 ± 1.896 *
log_10 _[LF (msec^2^)]	4.862 ± 0.480	4.862 ± 0.480	4.863 ± 0.478	4.863 ± 0.478	4.864 ± 0.478	4.858 ± 0.486 *
log_10 _[HF (msec^2^)]	4.931 ± 0.537	4.931 ± 0.537	4.932 ± 0.534	4.932 ± 0.533	4.936 ± 0.525	4.938 ± 0.521 *
%LF (%)	46.91 ± 20.01	46.89 ± 20.00	46.88 ± 19.95	46.88 ± 19.96	46.68 ± 20.06	46.33 ± 20.14 *
log_10 _[LF/HF ratio]	−0.069 ± 0.416	−0.069 ± 0.416	−0.069 ± 0.415	−0.069 ± 0.415	−0.073 ± 0.416	−0.081 ± 0.420 *

*Notes:* LF (low frequency) and HF (high frequency) powers were calculated by spectral analysis shown in Figure 2; * shows *p* < 0.05 of significance levels obtained by two-way analysis of variance (*F* test) adding data of a lower sampling-frequency band stepwise.
